# Supplementary material for: Epigenetically driven and early immune evasion in colorectal cancer evolution
Source: Nat Genet. 2025 Nov 5;57(12):3039–49. doi: 10.1038/s41588-025-02349-1 (PMC12695633; doi:10.1038/s41588-025-02349-1)
Supplement: Supplementary file 1 — Supplementary Note, Methods and Figs. 1 and 2. [file 41588_2025_2349_MOESM1_ESM.pdf]

nature genetics

Supplementary information https://doi.org/10.1038/s41588-025-02349-1

Epigenetically driven and early immune evasion in colorectal cancer evolution

In the format provided by the authors and unedited

TABLE OF CONTENTS

Supplementary Note	2
Supplementary Methods	4
Supplementary Figure 1	12
Supplementary Figure 2	13
References	14

SUPPLEMENTARY NOTE

Phylogenetic analysis of antigen presenting genes

In our prior work, we found that gene expression in CRCs shows high plasticity and low heritability[1]. Motivated by these findings, we examined the heritability of expression within APGs specifically (see Supplementary Note) using phylogenetic signal analysis[2,3]. Nine cancers had sufficient number of biopsies with matched WGS and RNAseq for this analysis. The strongest phylogenetic signal (correlation between expression and evolutionary distance) was that of HLA-A expression in patient C559, which had a clade of biopsies with subclonal LOH in HLA-A. TAPBP and HSP90AB1 were the only recurrently phylogenetic genes although we detected somatic mutations in neither (Extended Figs. 1 & 3d). However, in patient C559 we detected differential patterns of open chromatin between tumour regions B and C&D (region A had no ATAC-sequenced samples) in the regulatory region upstream of HSP90AB1 (Extended Data Fig. 3e), making it plausible that heritable epigenetic variation is behind expression regulation. The generally low level of phylogenetic signal (signal found only in 17/297 gene-cancer combinations evaluated), combined with high intra-tumour heterogeneity (expression differences on the magnitude of median expression) confirms that the expression of antigen presenting genes is plastic, similar to the majority of genes.

Gene expression regulation as a mean of neoantigen depletion

In our EPICC cohort, we had previously clustered genes into four gene groups according to each gene's expression level across and variability within cancers[1], where group 1 was the most highly and uniformly expressed, and group 4 consisted of lowly expressed genes. For each group, we compared the proportion of neoantigens to non-antigenic protein-changing mutations (neoantigen ratio) falling within these genes. Clonal neoantigen ratios were significantly lower in gene groups 1 and 2 (Extended Data Fig. 4b), while subclonal neoantigen ratios were similar for all gene groups (Extended Data Fig. 4c). Groups 1&2 showed a significant depletion of clonal SNV neoantigens (Fisher's exact test $OR_{(neoantigen\ \&\ in\ group\ 1)}$ =0.73[0.54-1.0] and $OR_{(neoantigen\ \&\ in\ group\ 2)}$=0.75[0.6-0.94], Fig. 2d). To confirm that this depletion was related to expression patterns, we identified consistently expressed genes in our cohort using the definition of Rosenthal et al.[4] (>=1TPM in >95% of the measured

tumour samples). Indeed, clonal SNV neoantigens were significant depleted within consistently expressed genes (Extended Data Fig. 4d). These results were re-iterated when analysing MMRd cancers alone (Fisher's exact test $OR_{(neoantigen\ \&\ in\ group\ 1)}$ =0.72[0.55-0.96]) and a similar, but non-significant trend was observed in MMRp cancers, likely due to low mutation numbers. Frameshift (FS) neoantigens, especially clonal FS mutations, showed an even stronger depletion in gene groups 1 and 2 and in consistently expressed genes (Extended Data Fig. 4e-h). Thus, clonal neoantigens are typically found in genes with low and/or variable expression.

Exploring intra-tumour distribution (sharedness) of neoantigens

Both proportional burden and immune-dNdS detect selection through the absence/decreased number of antigenic mutations, and therefore detects only selection that leads to mutation elimination. For contrast, we assessed neoantigen variant allele frequency (neoantigen-VAF) that can detect antigenic clones which are neoantigen depleted, but not eliminated, indicative of weak or ongoing selection[5]. While we did not observe consistent neoantigen-VAF depletion across (superficial) samples (Extended Data Fig. 8a-b), significant depletion was observed when analysis was restricted to escaped MMRp cases (Extended Data Fig. 8c, KS-test p=0.012). Similarly, neoantigens were as likely to be shared between samples from a tumour as non-antigenic mutations (Extended Data Fig. 8d-f).

SUPPLEMENTARY METHODS

Analysis of CyCIF images

One raw image file was acquired in the .ndpi format per cycle of scanning. Raw images were cropped using ImageJ[6] such that all images were centred on the region of interest (ROI) that was serial to that used for genomics at LCM. Images were cropped to the same width and height dimensions across cycles to enable registration. Differences in background illumination were corrected using the built-in rolling ball algorithm in ImageJ.

The MCMICRO pipeline[7] was used for registration (ASHLAR), segmentation (UnMICST/ S3segmenter) and signal quantification (MCQuant). Autofluorescence was removed by subtracting the intensity values noted for FITC and TRITC during the background scan (with no markers) from intensity values for markers in the first 3 marker cycles using R. Artefacts were identified by plotting the coordinates of cells showing expression values in the 99.95th quantile for each marker on the raw image (i.e. the cells with the highest expression) using R. If such cells were localised to an illumination artefact, then those cells were removed from all further analysis by specifying coordinates for the affected area.

For each sample, for each marker, coordinates of cells with expression above a range of quantiles were plotted serially on the raw image (as red dots) for that marker using R. Images were then reviewed manually: a quantile where red dots overlapped with most positive cells on the raw image with few false positives was selected as the quantile threshold for that marker. All markers for all samples were reviewed similarly to identify a quantile threshold. Any values lower than the quantile threshold were set to 0.0001 for each marker. After thresholding, for each marker, expression per cell was divided by the 99.99th quantile for that marker using R. This allowed us to scale expression for different markers to similar ranges to allow us to identify co-expression of markers using clustering.

Using R, coordinates of all cells identified using MCMICRO were plotted on a raw DAPI mask. The “clickpoly” function from the R package spatstat v2.3-4 was used to interactively draw a polygon around the ROI. The polygon was expanded by a small

amount of buffer region (50 units) to minimise any differences across ROIs in manually drawing the boundary. Only cells falling within the ROI were subsetted for further analysis.

The final list of 20 markers used for clustering was: Ki67, iNOS, CD45, CD8, IDO1, PD1, CD163, CD3, PDL1, CD4, CD68, VISTA, CD20, CTLA4, CD45RO, MYELO, ECAD, FOXP3, VIMENTIN, CK.

Expression of all markers for all cells from all CyCIF ROIs (1627807 cells in total) were clustered with R PhenoGraph, the R implementation for the PhenoGraph algorithm (R package: Rphenograph v0.99.1) with k=45. PhenoGraph takes as input a matrix of N single-cell measurements and partitions them into subpopulations by clustering a graph that represents their phenotypic similarity[8]. 262 clusters were identified. For each cluster, median normalised expression of every marker was identified. For a given cluster, if median normalised expression was greater than 0 for a marker, the cluster was considered positive for that marker.

32 clusters with no positive markers were excluded from further analysis. Such clusters may represent cells with signal below the threshold applied in previous steps for a marker (with each such cell showing marker expression 0.0001). 3 clusters showing positive markers that would not be expected together – i.e. a mixture of epithelial/ stromal/ immune markers – were also excluded. Otherwise, the following rules were used for labelling phenotypes, implemented in R:

Epithelial cells:

- Only CK/ECAD/ both: phenotype of clusters was labelled as “epithelial cells”.
- CK/ECAD/ both with Ki67: phenotype of clusters was labelled as “proliferating epithelial cells”.
- Clusters expressing ECAD/CK/both and Vimentin were labelled "Mixed epithelial/stromal" and excluded from further analysis.

Stromal cells:

- Only Vimentin: phenotype of clusters was labelled as “stromal cells”.

Immune cells:

- If only 1 immune marker was included, the phenotype was labelled as the marker followed by a “+” sign.
- Multiple markers:

- If 2 or more markers were present with all being immune markers, all makers were included in the final phenotype.
- If 2 or more markers were present with at least one immune marker and a second marker being “ECAD/CK/ Vimentin /CD45”, this additional second marker was ignored in the final phenotype. This was because:
    i. An assumption was made that mixed epithelial/ stromal component with immune markers was due to difficulties cleanly segmenting infiltrating immune cells from epithelial/ stromal background. In this situation, the immune marker was more relevant to the phenotype than the contaminating epithelial/stromal component.
    ii. As CD45 is the lymphocyte common antigen, it does not add additional distinguishing subtype-specific information. For example, clusters which are CD45+CD3+ and CD3+ only both show a phenotype of T cells (the subtype-specific marker is paramount in this case and both clusters would be labelled CD3+ for analysis purposes). Similarly, cluster 116 (showing CD45,CD8,CD3, cluster 254 showing CD8,ECAD,CK and 162 showing CD8 only were labelled as “cytotoxic T cells”.
    iii. Clusters expressing only CD45 or CD45 with epithelial/stromal component were labelled as "Lymphocyte, not otherwise specified".
- The rule under 4b for phenotyping of immune/epithelial clusters was not applied to PDL1 and CK/ ECAD as PDL1 may be expressed by epithelial or immune cells. Cluster 120 expressing PDL1 CK and 122 expressing PDL1 ECAD were both classified as “PDL1+ epithelial cells”.

After applying the above rules, 29 phenotypes were identified in total. 3 phenotypes were excluded for further analysis (“NA”, “Mixed epithelial/ stromal”, “PD-1+ MYELO+”), leaving 26 phenotypes for downstream analysis.

Visual assessment of phenotypes

For each ROI, coordinates of a subset of cells were plotted over the raw DAPI image used for segmentation to visually assess appropriateness of phenotyping. Phenotypes which were prevalent across ROIs such as epithelial cells, stromal cells, lymphocyte

(no specific type) and cytotoxic T cells were selected for visual assessment. All epithelial cell phenotypes ("Epithelial cells", "PDL1+ epithelial cells" and "Proliferating epithelial cells") were grouped together as "Epithelial cells". Additionally, the commonly prevalent immune markers of CD3/CD4/CD68/CD163/CD20/MYELO were selected such that any cells labelled with a cluster positive for any one of these markers would be included.

Where cells were labelled for more than one included marker, the marker pertaining to the broadest category of cells was used as the final label. For example, if a cell was positive for both CD3 and CD4, CD3 was prioritised as the label. Similarly, CD68 was prioritised over CD163. This was to allow easy visualisation of broad categories rather than niche subtypes.

Distance between epithelial and immune cells

The R package spatstat v2.3-4 was used to determine the pairwise Euclidean distances between cells of different phenotypes. For each ROI, cells of the two phenotypes of interest were identified and pairwise Euclidean distances established using the function crossdist(). For each cell of interest, the closest cell of the second phenotype was determined by taking the shortest pairwise distance of all cells of the second phenotype. The Euclidean distance in pixels was converted to micrometres by multiplying the distance by 0.44µm, the size of each pixel.

For this analysis, phenotypes "proliferating epithelial cells" and "epithelial" cells were considered together as epithelial cells (PDL1+ epithelial cells were not included here). All phenotypes that were positive for Ki67+ in addition to a cell type marker were clubbed together. In other words, phenotypes "Ki67+CD20+ and "CD20" were clubbed together, as were "Ki67+MYELO+" / "MYELO+" and "Ki67+PDL1+" / "PDL1+". Where all lymphocytes were considered together, they were grouped as described above.

Cellular neighbourhoods (CN)

We adapted the method of cellular neighbourhood classification from Schürch et al.[9]. For each of the 1,146,536 cells across all ROIs, a 'window' was captured consisting of the 10 nearest neighbouring cells (including the centre cell) as measured by Euclidean distance between X/Y coordinates. This was an unsupervised approach implemented through NearestNeighbors

(from sklearn.neighbours) with the “auto” algorithm (default). These windows were then clustered by their composition with respect to the 26 cell types that had previously been using Python’s *scikit-learn* implementation of MiniBatchKMeans with number of neighbourhoods k = 15. In this manner, 15 cellular neighbourhoods were identified, which were each enriched for a variety of cell types. Each cell was then allocated to the CN that its surrounding window was in. To validate the CN assignment, these allocations were overlaid on the original nuclear mask from the fluorescent images.

For each CN, for a cell type, the mean number of nearest neighbours matching this cell type represented in the CN was noted (“cluster “centroids”). For every cell type, the proportion of cells in this cell type out of all cells (from all ROIs) was established. This proportion was added to the cluster centroids for the matching cell type. The overall sum was divided by 11 (the sum of mean number of nearest neighbours (10 for each cell) and sum of proportions for all cell types (1) and was then divided by the proportion of cells for a cell type (as determined above) to normalise for cell type frequency. Based on the cell type composition for each CN, each CN was given a unique label. Every cell across all ROIs was assigned to a CN.

To compare immune cell subtypes within CNs, the 6 CNs positive for epithelial cells were identified, namely CN1: "Epithelial cell-enriched", CN0: "Tumour-immune interface I", CN5: "Tumour-immune interface II", CN14: "Tumour-immune interface III", CN3: "PDL1+ and PDL1-epithelial cell-enriched" and CN10: "Cells expressing ICRs, CD4+ and macrophage-enriched". CNs positive for PDL1+ epithelial cells (CN3 and CN10) were compared to the other CNs. Immune cell types were defined as all phenotypes other than “Epithelial cells”, “Proliferating epithelial cells”, "PDL1+ epithelial cells", “stromal cells” and “Ki67+ only”.

To compare specific cell types within CNs across sample types, the fraction and number per epithelial cell for each cell type of interest (e.g. PD1+, PD1+CD3+CD45RO+, PDL1+ and PDL1+ epithelial cells) within each CN in each ROI were identified and compared measures across sample types.

Ripley’s H Index

Ripley's H-index[10] was calculated using the Tumour Landscape Analysis pipeline (https://github.com/cisluis/TLA/blob/main/documentation/TLA_doc.md). For each reference cell, the number of test cells $I_{rt}(d)$ inside a radius d from the reference cell is established. The Ripley's K function is the mean across all reference cells normalized by the density λ_t of test cells (overall density across the entire ROI).

$$K_{rt}(x,y) = \frac{1}{\lambda_t}\langle I_{rt}(d)\rangle_{(x,y)}$$

K is effectively the proportion of observed to expected points in the circle surrounding a reference cell of radius d. When the distribution of test points is homogeneous (i.e. number of test cells within the circle is similar to number expected based on overall test cell density), (i) the expected value of I should approach A λ_t with A= πd^2 (the area of the circle); (ii) the expected value of K should approach A (with A= πd^2).

The *H* function is defined as:

$$H_{rt}(x,y) = \sqrt{\frac{K_{rt}(x,y)}{\pi}} - d$$

The H function is a measure of the level of clustering of test cells around reference cells at the scale *d*. When the number of test points within the circle is similar to number expected based on overall test cell density, K= πd^2, H=0. When the number of test points within the circle is lower or higher than expected, H becomes negative or positive, respectively. *H* ~ 0 indicates that reference and test cells are mixed uniformly. We used d=100 μm as interactions between immune cells and epithelial cells are likely to occur within this proximity – this metric has been used in previous work from our collaborators[165].

VAF distribution of neoantigens/non-antigenic mutations

First, we defined the unadjusted VAF of each mutation as the number of variant reads divided by the number of total reads spanning the locus (Platypus information NV/NR). Then we corrected this value to account for differences in purity: VAF = 1/purity * VAF_{unadj}. Purity values for FF-WGS samples were derived from sequenza following manual curation. Purity values for FFPE-PS samples were derived from deep learning classifier applied to the H&E images (see below) overlapping the site resected for sequencing.

Each mutation was annotated according to antigenicity. In FF-WGS samples, we denoted non-binders as non-antigenic mutations and strong-binders as antigens; in

FFPE-PS samples we used weak- and non-binders with recognition potential <0.1 as non-antigens and strong-binders with recognition potential >0.1 as antigens, but also explored weak- vs strong-binders (ignoring recognition potential). We only considered SNVs in this analysis. In order to overcome the low numbers of observed mutations, we pulled all mutations from a given set of samples (e.g. samples from escaped MMRp cancers). We then compared the distribution of antigenic and non-antigenic mutations using Kolmogorov-Smirnov test, and visualised both cumulative distributions against the inverse of VAF, looking for the previously established signal of immune selection[5]. In addition, we also evaluated the proportion of antigenic and non-antigenic mutations that were within a certain range of VAF, compared to all antigenic and non-antigenic mutations, respectively. We compared these proportions computed for each sample separately, using a paired Wilcoxon rank sum test.

Clonal mutation down-sampling

To carry out the down-sampling analysis in Extended Data Fig. 9, for each cancer we took the list of SNVs (i.e. within-gene, nonsynonymous point mutations), and constructed a new list by including all subclonal mutations and 25% of clonal mutations. Clonal/subclonal mutations were defined for CRAs and CRCs separately, therefore the same applied to shared mutations within a CRA. We then computed proportional neoantigen burden for each cancer as done for the full datasets, and ran multivariable regression and subclone-specific normalised burden analysis. Multivariable regression did not converge in 8/50 datasets due to the decreased mutation counts.

Phylogenetic analysis

Phylogenetic signal of antigen presenting gene expression was assessed following the procedure detailed in ref[1]. We kept only leaves of each tree that had matched RNA sequencing, including both deep and low-pass WGS samples. Then, we randomly assigned the branch lengths to low-pass WGS samples, and repeated this procedure 100 times to obtain a median estimate of phylogenetic signal, as described in detail by ref[1]. We limited the analysis to cancer samples and to cases that had at least 6 biopsies in the final pruned trees.

Phylogenetically close biopsies for the subclonal immune escape analysis (Fig. 5c&d) were defined using the following steps: (1) identify the branch on which the subclonal

mutation is located; (2) identify the closest branch without the mutation; (3) include the next level branches as well if less than three deep-sequenced samples are found on the original two branches. The set of samples considered for each cancer are shown in Extended Data Fig. 1.

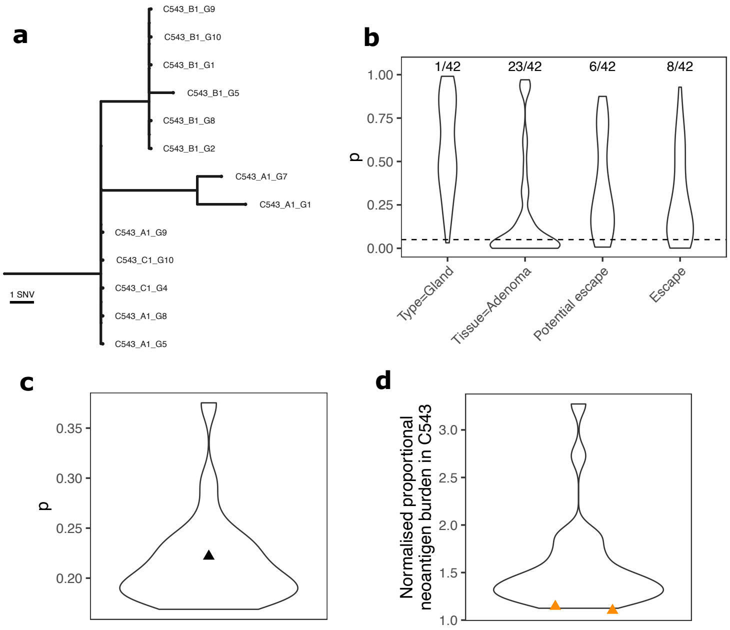

Supplementary Figure 1. Down-sampling analysis of clonal/subclonal mutations. (a) An example phylogenetic tree of C543 following down-sampling of clonal mutations, showing only SNVs. (b) Distribution of p-values of multivariable regression on proportional neoantigen burden (see Fig. 5a) obtained for each categorical variable. The number of significant associations (out of 42 converging regression analyses) are shown above each violin plot. P=0.05 is indicated by dashed line. (c) Distribution of p-values of t-test on normalised proportional burden of subclonally escaped biopsies (see Fig. 5c) in the 50 down-sampled datasets. Original p-value from full dataset is shown with black triangle. (d) Distribution of normalised proportional burden values in C543 in the 50 down-sampled datasets. Original values from full dataset are shown with orange triangles.

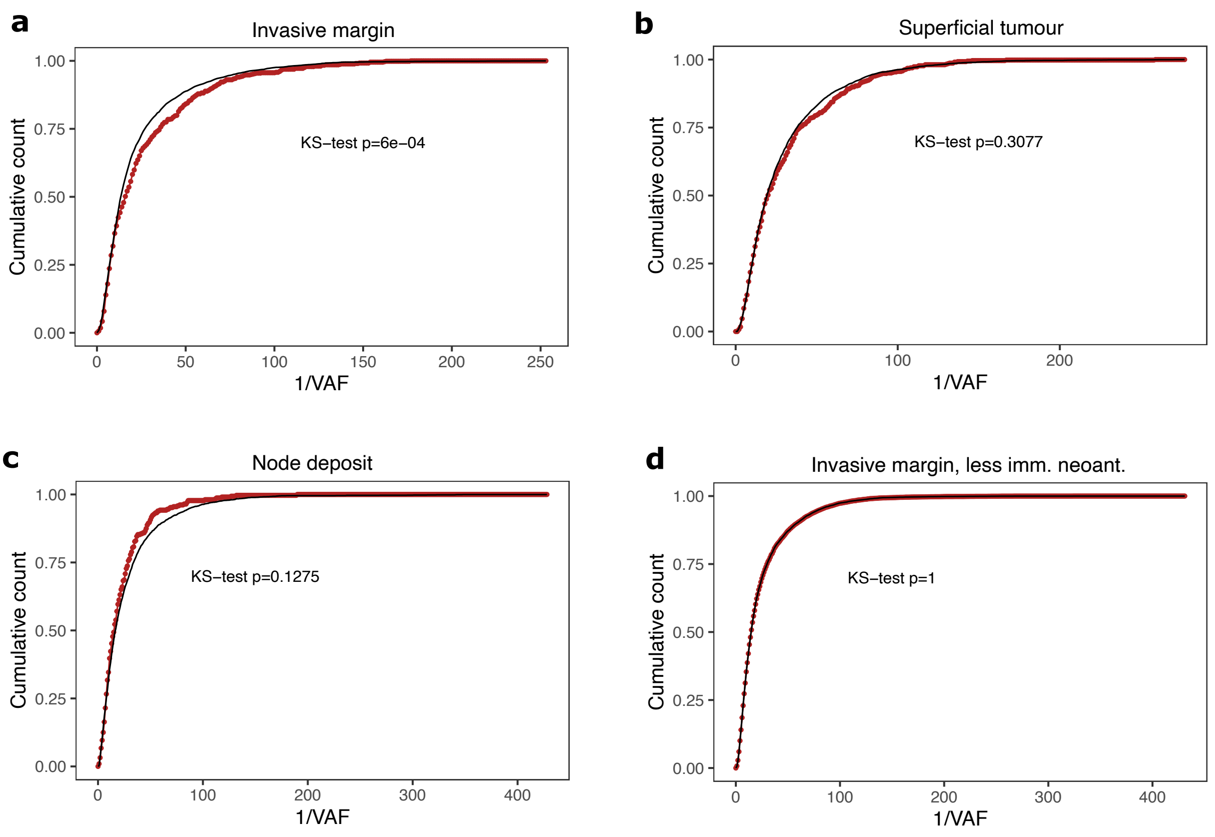

Supplementary Figure 2. Frequency distribution of neoantigens in different tumour sample types. The cumulative number of low-immunogenicity mutations (grey) and high-immunogenicity neoantigens (red) shown against the inverse of the variant allele frequency. All mutations were pulled together from FFPE-PS samples from the invasive margin (a,d), superficial tumour (b) or node (d). In (a-c), immunogenic neoantigens are defined as strong-binders with high recognition potential and compared to weak-binders with low recognition potential. In (d), they are defined as strong-binders and compared to non-binders.

REFERENCES

1. Househam, J. *et al.* Phenotypic plasticity and genetic control in colorectal cancer evolution. *Nature* **611**, 744–753 (2022).
2. Pagel, M. Inferring the historical patterns of biological evolution. *Nature* **401**, 877–884 (1999).
3. Freckleton, R. P., Harvey, P. H. & Pagel, M. Phylogenetic analysis and comparative data: a test and review of evidence. *Am. Nat.* **160**, 712–726 (2002).
4. Rosenthal, R. *et al.* Neoantigen-directed immune escape in lung cancer evolution. *Nature* **567**, 479–485 (2019).
5. Lakatos, E. *et al.* Evolutionary dynamics of neoantigens in growing tumors. *Nat. Genet.* **52**, 1057–1066 (2020).
6. Fiji: an open-source platform for biological-image analysis | Nature Methods. https://www.nature.com/articles/nmeth.2019.
7. Schapiro, D. *et al.* MCMICRO: a scalable, modular image-processing pipeline for multiplexed tissue imaging. *Nat. Methods* **19**, 311–315 (2022).
8. Levine, J. H. *et al.* Data-Driven Phenotypic Dissection of AML Reveals Progenitor-like Cells that Correlate with Prognosis. *Cell* **162**, 184–197 (2015).
9. Schürch, C. M. *et al.* Coordinated Cellular Neighborhoods Orchestrate Antitumoral Immunity at the Colorectal Cancer Invasive Front. *Cell* **182**, 1341-1359.e19 (2020).
10. Ripley, B. D. Modelling Spatial Patterns. *J. R. Stat. Soc. Ser. B Methodol.* **39**, 172–192 (1977).
