# Supplementary material for: Epigenetically driven and early immune evasion in colorectal cancer evolution
Source: Nat Genet. 2025 Nov 5;57(12):3039–49. doi: 10.1038/s41588-025-02349-1 (PMC12695633; doi:10.1038/s41588-025-02349-1)
Supplement: Supplementary file 2 — Reporting Summary [file 41588_2025_2349_MOESM2_ESM.pdf]

nature portfolio

Corresponding author(s): Eszter Lakatos
Andrea Sottoriva
Trevor Graham

Last updated by author(s): Jun 9, 2025

Reporting Summary

Nature Portfolio wishes to improve the reproducibility of the work that we publish. This form provides structure for consistency and transparency in reporting. For further information on Nature Portfolio policies, see our Editorial Policies and the Editorial Policy Checklist.

Statistics

For all statistical analyses, confirm that the following items are present in the figure legend, table legend, main text, or Methods section.

n/a	Confirmed	
☐	☒	The exact sample size (n) for each experimental group/condition, given as a discrete number and unit of measurement
☐	☒	A statement on whether measurements were taken from distinct samples or whether the same sample was measured repeatedly
☐	☒	The statistical test(s) used AND whether they are one- or two-sided<br>*Only common tests should be described solely by name; describe more complex techniques in the Methods section.*
☐	☒	A description of all covariates tested
☐	☒	A description of any assumptions or corrections, such as tests of normality and adjustment for multiple comparisons
☐	☒	A full description of the statistical parameters including central tendency (e.g. means) or other basic estimates (e.g. regression coefficient) AND variation (e.g. standard deviation) or associated estimates of uncertainty (e.g. confidence intervals)
☐	☒	For null hypothesis testing, the test statistic (e.g. F, t, r) with confidence intervals, effect sizes, degrees of freedom and P value noted<br>*Give P values as exact values whenever suitable.*
☒	☐	For Bayesian analysis, information on the choice of priors and Markov chain Monte Carlo settings
☐	☒	For hierarchical and complex designs, identification of the appropriate level for tests and full reporting of outcomes
☒	☐	Estimates of effect sizes (e.g. Cohen's d, Pearson's r), indicating how they were calculated

Our web collection on statistics for biologists contains articles on many of the points above.

Software and code

Policy information about availability of computer code

Data collection	No software was used for data collection.
Data analysis	The following software were used for sequencing data and image processing: NDPViewer (v2.9.29), Fgbio (v1.3.0), Picard (v2.20.3), Mutect2 (v4.1.4.1), Platypus (v0.8.1.1), polysolver (v1.0), MuTect (v1.16) within polysolver, Strelka2 (v2.9.10), sequenza (v2.1.2), LOHHLA (https://bitbucket.org/mcgranahanlab/lohhla/src/master/), SOPRANO (https://github.com/luisgls/SOPRANO), NeoPredPipe (https://github.com/MathOnco/NeoPredPipe), bam-readcount (v1.0.1), R package lme4 (v1.1.36)<br>See Methods section of the manuscript for further detail.. All data analysis script in R (v4.4.2) creating processed data and producing figures is available from https://github.com/elakatos/EPICC_immune_analysis .

For manuscripts utilizing custom algorithms or software that are central to the research but not yet described in published literature, software must be made available to editors and reviewers. We strongly encourage code deposition in a community repository (e.g. GitHub). See the Nature Portfolio guidelines for submitting code & software for further information.

Data

Policy information about availability of data

All manuscripts must include a data availability statement. This statement should provide the following information, where applicable:

- Accession codes, unique identifiers, or web links for publicly available datasets
- A description of any restrictions on data availability
- For clinical datasets or third party data, please ensure that the statement adheres to our policy

Processed data used in the figures and to derive summary tables are available at Mendeley: https://doi.org/10.17632/cjfmmc95dm.2. Raw sequencing reads of FFPE-PS samples are currently made available on the European Genome-Phenome Archive (please see our cover letter for details on the delay in providing accession codes). Raw sequencing data of FF-WGS samples is available at accession code EGAS00001005230, and previously generated processed data at https://data.mendeley.com/datasets/7wx3chtsxx/2.
For alignment of sequencing reads, reference genome hg38 accessible at https://ftp.1000genomes.ebi.ac.uk/vol1/ftp/technical/reference/GRCh38_reference_genome/ was used.

Research involving human participants, their data, or biological material

Policy information about studies with human participants or human data. See also policy information about sex, gender (identity/presentation), and sexual orientation and race, ethnicity and racism.

Reporting on sex and gender	Our work used re-analysis and re-sequencing of previously collected data. All investigators were blinded to clinicopathological information and all data analysis considered the dataset as a whole without accounting for patient characteristics.
Reporting on race, ethnicity, or other socially relevant groupings	See above.
Population characteristics	Our work used re-analysis and re-sequencing of previously collected data. For information on the original data, see information reported in https://www.nature.com/articles/s41586-022-05202-1 .
Recruitment	See above.
Ethics oversight	All patients gave informed consent for collection of their materials to the UCLH Cancer Biobank (Research Ethics Committee approval 15/YH/0311). All investigators were blinded to patient data related to outcome, and all clinicopathological information.

Note that full information on the approval of the study protocol must also be provided in the manuscript.

Field-specific reporting

Please select the one below that is the best fit for your research. If you are not sure, read the appropriate sections before making your selection.

☒ Life sciences ☐ Behavioural & social sciences ☐ Ecological, evolutionary & environmental sciences

For a reference copy of the document with all sections, see nature.com/documents/nr-reporting-summary-flat.pdf

Life sciences study design

All studies must disclose on these points even when the disclosure is negative.

Sample size	No sample size calculation was performed but all available data from our previous dataset was used. For FFPE sample analysis, all patients with stage III MMRp cancer with lymph node deposit were used. Post-hoc simulations to highlight shortcomings in statistical power were performed and included in the manuscript.
Data exclusions	RNAseq data were filtered based on paired WGS purity (>0.05) and read count (>5M reads). WGS data were filtered based on purity (>20%). Unless stated otherwise, colorectal adenomas were excluded from all RNAseq and WGS analysis. Wherever indicated in figure legend/title, MMRd carcinomas were excluded and analysis limited to MMRp CRCs. In downstream analysis of processed images, cells with contradictory positive markers were excluded.
Replication	No experiments, only data extraction and analysis were carried out in the study. Repeat sampling of FF-WGS/FFPE-PS tumours served as pseudo-replicates.
Randomization	Our study was observational, so randomisation was not relevant for the study setting. In all analysis, purity was taken into account as covariate. In multivariable regression, sample type (gland/bulk) and purity were both included as covariates. In all analysis, adenoma vs carcinoma label was either included as a covariate or adenomas filtered out prior to analysis.
Blinding	All investigators were blinded to all patient clinical characteristics.

Reporting for specific materials, systems and methods

We require information from authors about some types of materials, experimental systems and methods used in many studies. Here, indicate whether each material, system or method listed is relevant to your study. If you are not sure if a list item applies to your research, read the appropriate section before selecting a response.

Materials & experimental systems

n/a	Involved in the study
☐	☒ Antibodies
☒	☐ Eukaryotic cell lines
☒	☐ Palaeontology and archaeology
☒	☐ Animals and other organisms
☒	☐ Clinical data
☒	☐ Dual use research of concern
☒	☐ Plants

Methods

n/a	Involved in the study
☒	☐ ChIP-seq
☒	☐ Flow cytometry
☒	☐ MRI-based neuroimaging

Antibodies

Antibodies used

Ki67 (CST, #11882S), CD8 (Fisher, #53-0008-82), CD163 (Abcam, #ab218293), CD45RO (Biolegend, #304212), CD20 (Fisher, #15301990), E-cadherin (CST, #3199S), IDO1 (CST, #10312S), CD3 (Abcam, #ab208514), CD68 (CST, #79594S), CTLA4 (Abcam, #ab283489), HLA-ABC (Fisher, #15804219), FOXP3 (Fisher, #15588936), Pan CK (antibodies online, #bs-1712R-A555) , PD1 (Abcam, #ab201825), PDL1 (Abcam, #ab267563), Vista (CST, #92734S), CD57 (Miltenyi, #130-111-964), Myeloperoxidase (Abcam, #ab252131), Vimentin (Biolegend, #677807), iNOS, CD45

Validation

The specificity of all primary antibodies was validated by a pathologist using FFPE sections of colorectal cancer. Antibodies were first tested in single-plex chromogenic immunohistochemistry, before incorporating in the multiplex CyCIF panel. At least three dilutions of each primary antibody were tested in multiplex CyCIF, and the dilution with the brightest specific signal with no non-specific signal was selected.

Plants

Seed stocks

Report on the source of all seed stocks or other plant material used. If applicable, state the seed stock centre and catalogue number. If plant specimens were collected from the field, describe the collection location, date and sampling procedures.

Novel plant genotypes

Describe the methods by which all novel plant genotypes were produced. This includes those generated by transgenic approaches, gene editing, chemical/radiation-based mutagenesis and hybridization. For transgenic lines, describe the transformation method, the number of independent lines analyzed and the generation upon which experiments were performed. For gene-edited lines, describe the editor used, the endogenous sequence targeted for editing, the targeting guide RNA sequence (if applicable) and how the editor was applied.

Authentication

Describe any authentication procedures for each seed stock used or novel genotype generated. Describe any experiments used to assess the effect of a mutation and, where applicable, how potential secondary effects (e.g. second site T-DNA insertions, mosiacism, off-target gene editing) were examined.
